# Evaluation of the factors influencing the housing safety awareness of residents in Shanghai

**DOI:** 10.1371/journal.pone.0227871

**Published:** 2020-01-24

**Authors:** Jin Ban, Longzhu Chen

**Affiliations:** Department of Civil Engineering, Shanghai Jiao Tong University, Shanghai, China; Tongii University, CHINA

## Abstract

Shanghai has experienced rapid urbanization and has a serious housing aging problem. The situation of urban housing safety management needs to be strengthened. However, in China, housing safety management (HSM) is just in its beginning stage and it lacks thorough research. Housing safety awareness is one of the most significant aspects of housing safety management. Therefore, in order to investigate the housing safety awareness of Shanghai residents, this paper investigates the safety attitudes of residents living in housing of different ages using consulting questionnaires and Statistical Package for Social Science (SPSS) software. The results show that in Shanghai, the residents lack an understanding of housing management law, policy, and awareness of safety use and have low willingness to buy commercial insurance. Based on these results, the factors that affect the safety awareness of Shanghai residents are summarized as follows: (1) asymmetric information; (2) assessment of the safety status of the premises; and (3) differences in house users.

## Introduction

By 2018, the construction area of Shanghai as the largest municipality in China had reached 672 million square meters [1]. However, some of the constructed buildings have various security problems [2] and are in urgent need of repair. Although the area of new buildings in Shanghai is very large, the reconstruction of the old city is also increasing. With the gradual development of mature urbanization, urban construction is becoming increasingly refined [3]. The pace of renovation of the old areas has gradually slowed down [4–5], as some high-density old houses are hard to remodel. The city still has a large number of old houses with hidden dangers, causing the housing safety problem to become increasingly serious. Different from the advanced degree of HSM in developed countries, HSM gradually came into the Chinese’ vision, until the Ministry of Housing and Construction issued a “Notice on Organizing the Safety Investigation of Old Building Dangerous Buildings” in China because of the residence buildings that collapsed in Fenghua City, Zhejiang Province which caused great loss of life and property on 4 April 2014 [6].

Shanghai has promulgated a series of measures for management at the housing using stage [7]. The safety management of housing is generally controllable, but some outstanding hidden dangers of housing safety still exist due to the large differences in the current security situation [8]. Domestic housing security research mostly focuses on the key technologies [9] and the risk managements of housing safety management [10–11]. Jiao [12] combined the theory of risk management with the safety management of buildings, identified and evaluated the risk of a building in its life cycle, and put forward corresponding control measures for various risk factors. Since then, the evaluation of risk has been gradually applied to the urbanization management of Shanghai. The research mostly focused on evaluation of disaster risk and less on social risk management (SRM). Studies included urban flood-risk assessment [13], promising directions for risk management [14], system for risk management based on the evaluation of safety risk for underground engineering [15] and the evaluation of challenges in landslide risk management to housing developments [16]. In addition, the research focuses on social risk management, especially residents' behavior is scarce. Shan [17] put forward strategies for risk management in the urban–rural conflict in urbanizing China. Yu [18] developed a model for managing social risks during the housing demolition stage of urban redevelopment projects and investigated the linkages between social risks and stakeholders. Compared with Hong Kong in the process of housing safety management, a series of studies based on the questionnaire and factor analysis method have been carried out on residents’ behavior, such as the impact of collective behavior of residents on housing safety management [19–21], while fewer studies have been conducted in Mainland China.

In addition to these risk management studies, ordinary residents are not familiar with housing safety management; therefore, it is necessary to consider residents' acceptance of policies. This study is conducted by dividing the housing age into samples of “more than 25 years” and “less than 25 years”; a survey of the residents' housing safety attention in Shanghai was carried out where the results are analyzed for residents' housing safety awareness. The influencing factors of the level of awareness were also explored in order to improve the level of housing safety management in Shanghai.

## State of building maintenance and safety management in Shanghai

### Problems of building maintenance in Shanghai

Many old residential buildings which have many security problems, were built in the1980s-1990s large-scale housing construction boom because of political and economic problems at that time. These houses have many safety problems that persist today. Historical reasons include the urbanization process of Shanghai [22].

- Before 1949: Before liberation
- 1950–1960: Restore the suburban industrial area built
- 1960–1980: Stagnation
- 1980–1990: Shanghai started large-scale housing construction.
- 1990–2000: Shanghai expanded to the outskirts of the city.
- 2000-Now: Effective management is needed

In Table 1, it can be seen that the proportion of seriously damaged and suspected dangerous houses is only 1.85%. This shows that the overall safety situation of existing aging houses in Shanghai is fine. However, due to many changes in design standards, poor construction quality, old age and other f actors, the safety status quo is very different leading to some hidden dangers of housing safety. The old houses in Shanghai mainly have the following safety problems.

**Table 1:** Old housing security screening in Shanghai in 2014.

Type	Area (million square meters)	Percentage (%)
**A. Years**		
1. More than 25 years	14,570	97.7
2. Less than 25 years	343	2.3
**B. Condition**		
1. Safe	1,575	90.3
2. General damage	7,017	7.84
3. Suspected serious damage	310	1.79
4. Suspected danger or Part of dangerous	9.6	0.06

### Design and construction problems

There was a period of stagnation before the 1980s because of the particularity of the Chinese economy and the political situation. It was not until the 1980s that there was a massive construction boom in China to reduce housing tension [23]. However due to the limitations of economic conditions the houses are both small and unreasonable layout. At the same time, due to some defects in the building standards [24], the quality of the house and the rationalization of residence are very low and persist to this day. After the 1980s, due to the Reform and Opening up program and the accumulation of social wealth, Shanghai reduced the number of cases of cutting corner during construction.

### House aging and lack of maintenance

For old houses, many buildings are in their overhaul and equipment renewal years. Old houses have a long construction period that accelerated aging. Private house owners neglect regular maintenance, and even if they find problems, they are not willing to repair them in time, leading to serious structural problem.

### Unsafe use building

In China, the owners are responsible for the lack of maintenance of their houses. In reality many homeowners usually emphasize the rights they should enjoy, rather than the responsibility and obligation to improve the performance of housing security. Some owners, in order to meet their living requirements, adopt blind transformation and violent decoration and neglect the safety of the building structure. Through the investigations of the Shanghai Building Quality Monitoring Stations in recent years, the number of projects due to changed structure housing security problems increased annually. In 2013, a total area of approximately 200,000 square meters of housing underwent renovation, which consisted of transformation (40%), decoration (20%), floor addition (10%) and other (30%) [25].

### Construction of houses

In recent years, the houses in central Shanghai have been greatly affected by the adjacent construction. The surrounding houses suffered damage such as uneven settlement and wall cracking, which are usually caused by subway construction, tunneling, deep foundation pits and municipal pipe networks [26–27]; other situation include municipal construction such as network management and channel damages to the housing situation, mainly in suburban county regions [28]. Data from the Shanghai Housing Quality Monitoring Station shows that in 2012 and 2013, the area of houses with problems caused by the construction of deep foundation pit was more than 300,000 square meters, which was approximately 15% of the total area of houses detected by the station. From 2011 to 2013, there were 26 projects for housing detection due to the influence of subway construction, covering an area of 1.328 million square meters [25].

### Present state of building safety management in Shanghai

**Fig 1:**
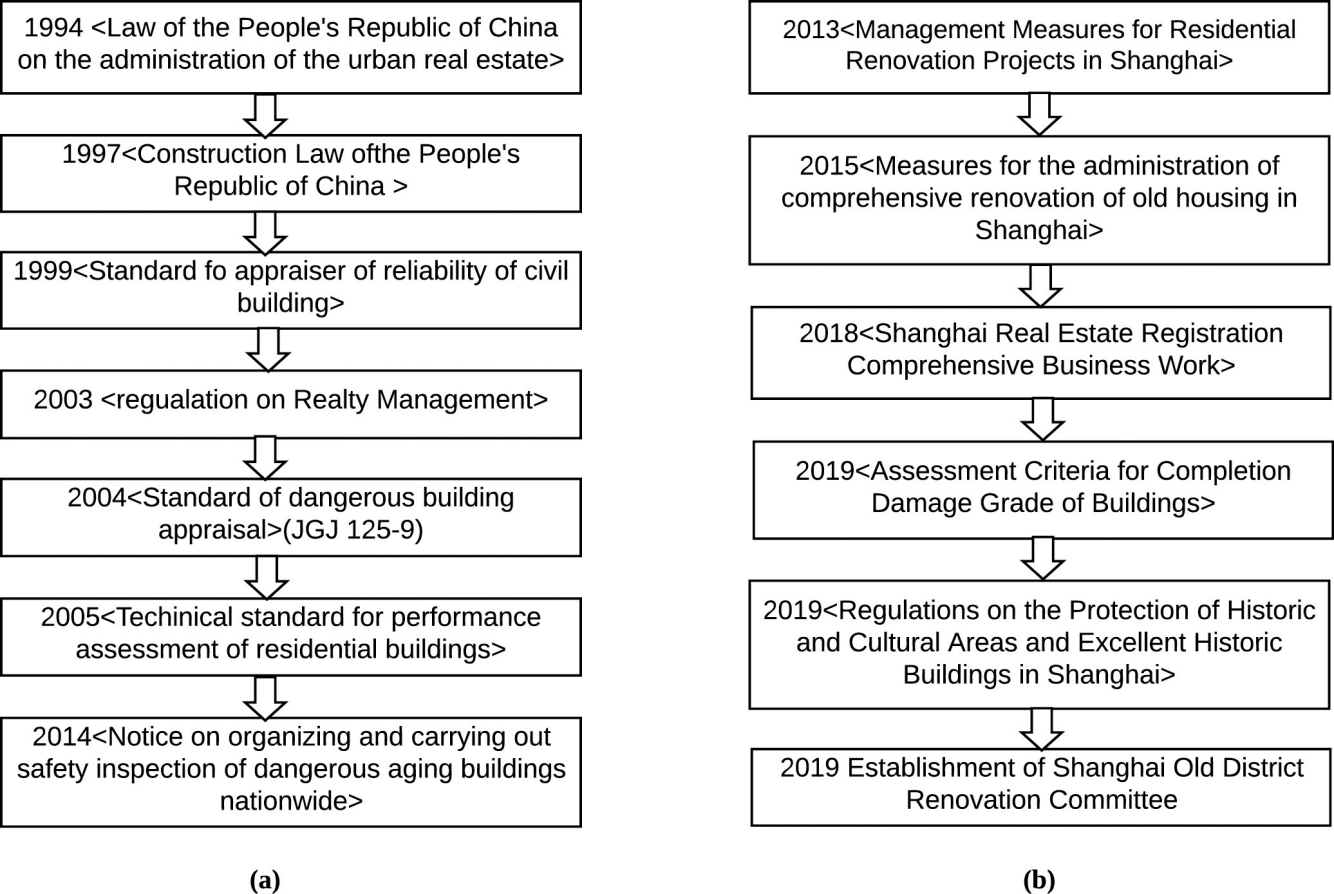
**(a)** Description of China's housing security management progress. **(b)** Description of Shanghai's housing security management progress.

We can see that the Shanghai housing security management focuses on comprehensive renovation of old housing and outstanding historical building protection (Fig 1). No broader and general policy has been introduced for ordinary houses. The housing safety management system is still in its infancy in Shanghai, while foreign research on housing safety management started earlier. Each stage of housing use is equipped with relevant standards or regulations to control the safety of the whole life cycle of housing. This is the case for countries such as Japan, the United States, the Soviet Union, Britain, Canada and others where building safety evaluation and reinforcement of publications were published earlier than in China [29–31]. At the same time, they have established corresponding organizations and relatively perfect housing safety management systems, which involve elements and methods of building safety performance evaluation.

## Methodology

The main housing problems mentioned in the previous chapter for Shanghai have limited considerations for residents during the design and construction stage. Therefore, this questionnaire focuses on three aspects: aging maintenance, safe use and surrounding safety. The questionnaire first invited Shanghai residents to a preliminary interview. In the presurvey, residents' safety awareness is mainly reflected in the cost, decoration and use of relevant housing information. Through the preinterview, 10 issues related to residents' concern about housing safety were summarized. By using the method of questionnaire survey and through the analysis of residents' awareness of housing safety, the safety attitudes of respondents under different grouping conditions were obtained. Each question was prepared according to the Likert scale method (scored from 1–5, where 1 is the lowest and 5 is the highest), where 1 = represents strongly disagree, 3 = agree, and 5 = strongly agree.

### Data collection

The area of apartment is approximate 92.8% of the total residential area in Shanghai [1]. In this questionnaire survey, the target respondents were residents living in apartments in Shanghai and were randomly selected to investigate Shanghai residents' safety awareness, mainly of them were from the Internet, a few of them were from distribution by hand. Any residents who were interested in the survey could answer the questionnaire. We received 1,949 questionnaires, including a total of valid 1,841 questionnaires, by means of distribution by hand (10) and online (1831).

In this questionnaire, the age samples were divided into four groups: “under 30”, “30–44”, “45–59” and “over 60”, as indicted in Table 2. Young people refer to those younger than 30 years old and those between 30 and 44 years old. Among the 1,841 valid questionnaires returned, young people accounted for more than 91% of the respondents, as shown in Table 2. This shows that there were fewer middle-aged and elderly people who are concerned with building safety, compared to young people.

**Table 2:** Background profiles of the survey respondents.

Information about respondents	Number of respondents	Percentage
A. Age		
1. Less than 30	839	45.7%
2. 30–45	843	45.9%
3. 46–60	141	7.7%
4. More than 60	13	0.7%
**Total**	**1,836**	**100%**
B. Type of housing age		
1. Less than 25 years	1,628	88.7%
2. More than 25 years	208	11.3%
**Total**	**1,836**	**100%**
C. Type housing acquisition		
Allocation	264	14.4%
Buy	1,036	56.4%
Rent	536	29.2%
**Total**	**1,836**	**100%**

According to the current situation in Shanghai, housing acquisition can be divided into three categories: rent, purchase and unit allocation (few in number), shown in Table 2. According to the data from 2017, the area of houses purchased and leased in Shanghai is basically the same [32] (No “Distribution Acquisition" Housing Data). However, 536 out of 1,836 responses were for rental housing, 1,036 for purchase and 264 for distribution which shows that residents who buy houses are more interested in housing safety management.

The repair cycle is divided into 25 years as ruled by the <Shanghai Technical specification for repair construction of buildings> DG-TJ08-207-2008. This questionnaire divided respondents’ residential houses into those within 25 years and more than 25 years, where 1,628 responses belonged in the “0–25 years” category and 208 belonged in the “more than 25 years” category. Since all the respondents’ age distribution, housing types, and living room age have universality, the participants' feedback and comments were reliable and representative. SPSS was then used to process the questionnaire data.

### Data analysis

**Fig 2:**
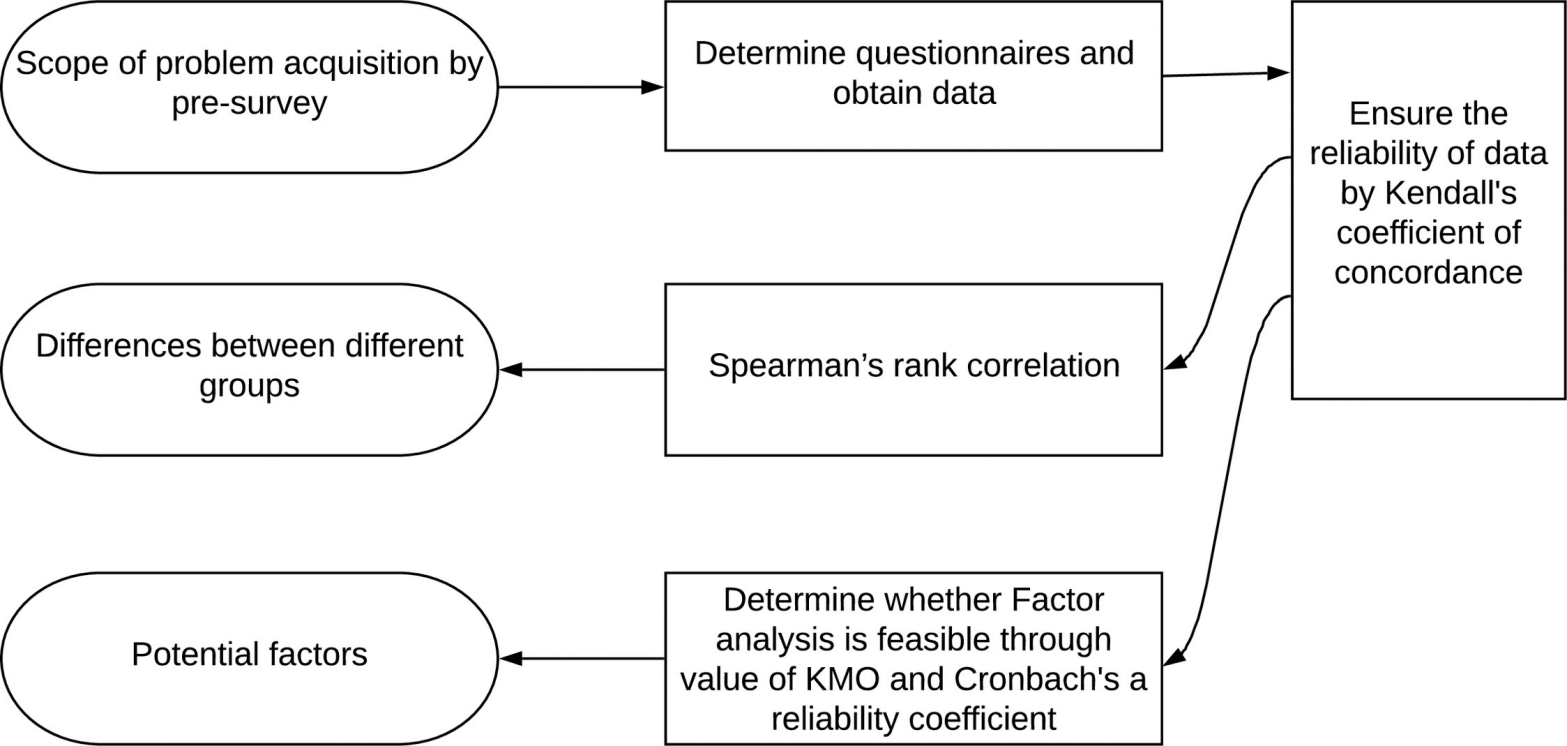
Data analysis flow chart.

To obtain useful information such as potential factors and differences between different groups, choosing appropriate method of data analysis is important [33]. First, Kendall's coefficient of concordance was used to test the agreement of respondents within each survey group to determine the consistency of data and ensure the reliability of data before proceeding to the next step [34]. Then, the overall ranking of the housing-safety awareness was carried out through Spearman's rank correlation to analyze the differences between different groups [35]. Finally, principal component analysis (PCA) was used as factor analysis method to explore the potential influencing factors [36], as shown in Fig 2.

## Results and analysis

First, Cronbach’s alpha coefficient was used to test the reliability of the questionnaire data [37]. The value of Cronbach’s alpha coefficient is between 0 and 1. If the alpha coefficient is less than 0.6, it is considered that the reliability of internal consistency is insufficient; from 0.7 to 0.8 indicates that reliability; and from 0.8 to 0.9, indicates that the reliability of the scale is very good. Through SPSS analysis, the Cronbach’s alpha coefficient of 10 groups of security awareness scoring questions is 0.747. Therefore, the data used in this questionnaire to measure Shanghai residents' awareness of housing safety are reliable, and the data from this questionnaire are reliable and can be analyzed in the next step.

Ten important issues of Shanghai residents' awareness of housing safety were analyzed from two groups, namely, “0–25 years of housing age” and “over 25 years of housing age”. The average score and ranking of each question in the total grouping and two different grouping samples are summarized in Table 3.

**Table 3:** Results of the ranking and Kendall's concordance test for the major building-safety awareness.

No.	Housing-safety awareness	All respondent group		0-25years group		More than 25 years group	
		mean	rank	mean	rank	mean	rank
2	Attention to Housing Safety Documents.	3.69	1	3.69	1	3.68	1
9	Degree of willingness to participate in the training of relevant knowledge of housing safety management.	3.48	2	3.48	2	3.45	2
3	Attention to the surroundings of houses.	3.43	3	3.43	3	3.45	3
1	Attention to the structure of residential buildings.	3.37	4	3.39	4	3.18	4
10	Demand for housing problem solving.	3.32	5	3.34	5	3.15	5
8	Degree of Demand for Legal Policies of Housing Safety Management.	3.29	6	3.3	6	3.14	6
5	Degree of willingness to appraise houses at one's own expense.	3.24	7	3.27	7	2.98	9
6	Degree of willingness to buy commercial housing insurance.	3.22	8	3.25	8	3.03	8
4	Degree of willingness to use houses safely.	3.06	9	3.07	9	3.05	7
7	Degree of understanding of housing management law and policy.	2.34	10	2.36	10	2.19	10
Number (N)		1,836		1,179		208	
Kendall's coefficient of concordance (W)		0.103		0.102		0.121	
Actual calculated chi-square value		1,701.887		1,488.663		227.199	
Critical value of chi-square from table		22		22		22	
Degree of freedom (df)		9		9		9	
Asymptotic level of significance		0.000		0.000		0.000	

H0 = Respondents' sets of rankings are unrelated (independent) to each other within each group.

Reject H0 if the actual chi-square value is larger than the critical value of chi-square from table.

Note: Items were rated on a 5-point Likert scale (1 = Strongly Disagree; 3 = Neutral and 5 = Strongly Agree).

### Agreement of respondents within each survey group

To assess the agreement of the “0–25 years group” and “more than 25 years group”, as shown in Table 4, the Kendall's coefficient of concordance (W) for the rankings of housing-safety awareness was 0.103, 0.102, and 0.121 for the “All respondent groups”, “0–25 years group” and “more than 25 years group”, respectively. The computed Ws were all statistically significant with a significance level of 0.000.

**Table 4:** Spearman's rank correlation coefficient.

Comparison of rankings	r_s_	Kendall's coefficient of concordance (W)	Significance level	conclusion
	0.0952	0.892	0.000	Reject H_0_ at 5% significance level

Since the number of attributes considered was more than seven, we used the chi-square value rather than the W value [38–41]. According to the degree of freedom (10–1 = 9) and the allowable level of significance (5%), the critical value of chi-square from the table should be 23.70 [42]. We then drew the hypothesis that the respondents' sets of rankings are independent of each other within a certain group. The actual computed chi-square values were 1,701.887, 1,488.663, and 227.199 for “All respondent group”, “0–25 years group” and “more than 25 years group”, respectively (Table 4) and were all larger than the critical value of chi-square of 23.70. This result indicates that the hypothesis has to be rejected. Consequently, it can be concluded that there is a significant degree of agreement among the respondents within each survey group and all respondents on the building-safety awareness of residents in Shanghai. In this paper, for the different respondents, the analysis of safety consciousness is consistent. The results show that the data collected from the questionnaire survey are valid and consistent for further analysis.

### Overall ranking of the building-safety awareness

From Table 3, the mean values range from 2.34 to 3.69 for all respondents. For those respondents living in a less than-25-year-old house, the mean values ranged from 2.36 to 3.69, while those rated by respondents living in more than 25-year-old houses, the mean values spanned from 2.19 to 3.68. Except for Item 3, the mean values of the other 9 items scored by respondents of the “less than 25 years” group are higher than that of the “more than 25 years” group. Hence, the respondents living in old buildings have a lower housing safety degree. The difference of mean values for the “less than 25 years” group (3.69–2.36 = 1.33) is smaller than that of the “more than 25 years” group (3.68–2.19 = 1.49), reflecting that residents living in old houses have higher safety consciousness than the residents living in new houses. The mean values in 9 out of 10 questions are 3.0 and are close to "agree", while those rated by respondents living in houses more than 25 years old is 8 out of 10. As a result, most respondents to the security question have certain safety awareness.

The bottom four options with the most serious problems are as follow: Item 5 “Degree of willingness to appraise houses at one's own expense”, Item 6 “Degree of willingness to buy commercial housing insurance”, Item 4 “Degree of willingness to use houses safely”, and Item 7 “Degree of understanding of housing management law and policy”.

Item 7, “Degree of understanding of housing management law and policy”, was ranked with the lowest score option (mean value = 2.34). The current law has relevant regulations for the management and maintenance of residential buildings. However, in general, these laws are just for home use and some stages in the process of building safety class norms and constraints. House collapses usually happen in dangerous old houses, therefore, more policies focus on the detection and investigation of dangerous houses. There are no complete laws and regulations for the life cycle which need to be further improved. At the same time, the identification of the responsible person after the accident is not clear enough. Only the government could bear the loss of a housing accident, which cannot guarantee housing safety management.

Item 4, “Degree of willingness to use houses safely”, was ranked as the second lowest awareness by all respondents (mean score of 3.06). Historically, China built a large number of houses for which the housing design layout is extremely unreasonable in order to meet the housing needs in 1980s. To pursue comfortable living, residents of old houses would be more willing to demolish and renovate to meet their own needs. The willingness of old house residents to use reasonable security levels (mean score of 3.05) is lower than that of the new housing residents (mean score of 3.07).

Item 6, “Degree of willingness to buy commercial housing insurance”, was ranked as the third bottom awareness by the “All respondents group” (mean score of 3.22). Housing commercial insurance is just emerging in China, and since few people know about it, few people purchase insurance. There are some insurances of housing safety in China, most of them only cover steel-concrete and brick-concrete structures of residential houses, and does not involve commercial houses. The cost is 80 / year, while the compensation amount is high. The main contents include: compensation for the main body of the house (200,000–20 million yuan), follow-up decoration (50,000–2 million yuan), property loss in the house (20,000–1 million yuan), water pipe safety (10,000–200,000 yuan) and life safety loss of residents (10,000–300,000 yuan) caused by accidents.

The life cycle of a building during the expense period is divided into three parts: warranty, warranty after normal use within the time limit, and meet the design life. Developers and builders should hold responsibility during the warranty period. During the warranty period and within normal service life, the owner should bear most of the cost. The insurance company taking enterprise profits first is more sensitive to the problems of the quality of the housing construction. In the use stage, the insurance company can evaluate housing insurance according to the situation of homeowners’ use of home decoration.

Item 5, “Degree of willingness to appraise houses at one's own expense”, was ranked the fourth bottom awareness by the “all respondents group” (mean score of 3.24). The level of willingness to appraise houses at one’s own expense (mean score of 2.98) is much lower for residents of old houses than for those who live in new buildings (mean score of 3.27). The security issues of old buildings are more serious than those in new buildings; however, because of high reinforcing fees, old housing residents are unwilling to appraise the house at their own expense. Cost is an important factor affecting new and old housing residents’ safety awareness.

### Comparison of survey responses between the “0–25 years group” and “more than 25 years group”

Having tested the internal consistency of the rankings within the respondent groups in the previous section, the next stage of analysis was to test whether there is any significant agreement or disagreement in the rankings of various safety awareness issues between the survey groups. This can be indicated by the Spearman’s rank correlation coefficient (rs); the results are shown in Table 4.

The Spearman’s rank correlation coefficient of the rankings between the “0–25 years group” and the “more than 25 years group” for building safety awareness was 0.892 with a significance level of 0.000, as shown in Table 4. Therefore, the null hypothesis has to be rejected. It can be concluded that there is significant correlation between the “0–25 years group” and the “more than 25 years group” on the rankings of housing safety awareness.

In particular, 8 items out of the total received the same ranks. The different rankings of safety awareness between old and new houses are as follows: Item 4 “Degree of willingness to use houses safely” and item 5 “Degree of willingness to appraise houses at one's own expense”. The awareness of the residents in an old house to use the house safely is higher than that of residents in a new house, and the willingness to appraise houses is lower.

### Factor analysis of the building-safety awareness

Factor analysis investigates the structure of interrelationships between the large numbers of variables by identifying a set of potential factors [43–45]. Principal factor extraction with promax rotation [46–47] and Kaiser Normalization [48–49] was launched through the SPSS factor program on the 10 housing safety questions.

The conditions of use should be met before the use of factor analysis. The first is the sample size. The sample size should be sufficient to carry out factor analysis as it complies with the ratio of 1: 5 for the number of variables involved to the necessary sample size [50–51]. The sample size should be more than 10 questions multiplied by 5 samples required for each factor, which means that at least 50 samples are required in order to meet the requirement of sample size. The number of samples collected in this study is 1,836, which met the requirement of sample size. The second condition is that the value of KMO (Kaiser—Meyer—Olkin) should meet the requirements of Table 5 [52]. The KMO value of this questionnaire was 0.882, which shows a “Good” degree of common variance and is well above the acceptable threshold of 0.5, implying that there is good internal consistency in terms of correlations among the 10 questions and that the adopted measurement scale is reliable, as shown in Table 6.

**Table 5:** Acceptance level of the KMO value.

KMO value	Degree of common variance
0.90–1.00	Excellent
0.80–0.89	Good
0.70–0.79	Middling
0.60–0.69	Mediocre
0.50–0.59	Poor
0.00–0.49	Unacceptable

**Table 6:** KMO and Bartlett’s test.

Kaiser-Meyer-Olkin (KMO) measure of sampling adequacy	Bartlett’s test of sphericity		
	Approximate χ^2^ value	df	Sig
0.882	9867.160	45	0.000

The 10 questions in this questionnaire were subjected to principal component factor analysis by SPSS. In brief, we chose the dimension reduction—factor analysis, set the rotation mode as the maximum variance method, and set the numerical absolute value choice to 0.4; the results are shown in Table 7. The three factors can be named as follows: “Asymmetric information”, “Assessment of the safety status of the premises” and “Differences in house users” according to the various items involved in the problems.

**Table 7:** Factor structure of principal factor extraction and promax rotation on the 10 housing safety awareness questions.

No.	Housing-safety awareness	Factor loading	Eigenvalue	Percentage of variance explained	Cumulative percentage of variance explained
**Factor 1. Asymmetric information**					
1	Attention to the structure of residential buildings.	0.869	4.806	32.941	32.941
10	Demand for housing problem solving.	0.846			
8	Degree of Demand for Legal Policies of Housing Safety Management.	0.841			
9	Degree of willingness to participate in the training of relevant knowledge of housing safety management.	0.838			
**Factor 2. Assessment of the safety status of the premises**					
3	Attention to the surroundings of houses.	0.862	1.220	24.357	57.298
2	Attention to Housing Safety Documents.	0.821			
4	Degree of willingness to use houses safely.	0.810			
**Factor 3. Differences in house users**					
5	Degree of willingness to appraise houses at one's own expense.	0.764	1.042	13.388	70.686
7	Degree of understanding of housing management law and policy.	0.663			
6	Degree of willingness to buy commercial housing insurance.	0.503			

### Factor 1—Asymmetric information

Factor 1 consists of four housing safety awareness questions: building structure, solve method of safety problems, demand for legal policies and willingness to learn more. It can be seen that with the safety situation of the house itself and safety problems arising while the house is occupied, the relevant housing safety management policies such as maintenance cycle information are not enough. Due to the lack of basic knowledge and relevant information sources of self-management, the residents did not follow the rules to ensure the safety of the house. Residents determine the hidden dangers of housing safety in time and do not know how to deal with them, which leads to the occurrence of housing safety accidents.

From the angle of policy implementation, the information is not transparent; for example the housing security information query and the feedback processing results about home safety complaints are difficult to see in the housing use stage. The asymmetry of information makes occupants take a negative attitude towards housing safety, and they are unwilling to assume too much responsibility for the safe use of housing. In China, the information for complaints and reports is not transparent enough, and there are even suspicions that law enforcement agencies and the accused cover for each other. Therefore, the regulatory mechanism should be more transparent, improving the degree of information disclosure of law enforcement units. After complaints and reports from surrounding residents, law enforcement agencies should investigate and notify illegal owners of rectification as soon as possible, and return the results to surrounding residents for continued supervision.

### Factor 2—Assessment of the safety status of the premises

This factor comprises three items that are related to safety factors of houses: safety around the house, house safety documents and safe use of houses. Residents are expected to know the hidden problems that have happened and may happen to the house, what problems have happened to the house and whether there are any records for these three points. If there are problems with the house or potential safety hazards around it, the vigilance of the residents’ increases and the safety awareness will be improved; if there are no safety problems or safety accidents around the houses, then the residents' safety vigilance is low and their awareness of the safety of the houses is greatly reduced. Therefore, building safety risk factors greatly affect the level of resident housing security awareness in Shanghai.

### Factor 3 –Differences in house users

This factor consists of three items that focus on the expense of housing safety, including “Degree of willingness to appraise houses at one's own expense”, “Degree of understanding of housing management law and policy” and “Degree of willingness to buy commercial housing insurance”. A large number of respondents said that they were not clear about what the specific housing safety management is and what measures should be taken, including inspection and maintenance, commercial insurance and so on. As a result, residents' willingness to inspect and repair their houses at their own expense and to purchase commercial insurance for housing safety is low. On the whole, the policy popularization of housing safety management still has a long way to go, and it needs to increase in popularization among the people.

Home repair maintenance and testing requires a certain cost. Building maintenance policy is divided into corrective, preventive and condition-based strategies [53]. However, in reality, housing renovation involves complex relationships among property, owners and adjacent owners. Many owners are unwilling to pay for renovation or testing, and either strategy is difficult to carry out. This situation requires some compulsory measures to be enacted by law. At the same time, the government should develop a variety of funding channels to solve the problem of funds.

The warranty period of newly built houses usually refers to the guarantee period promised by the construction unit to the occupants of the houses, which is usually short [54]. Upon expiration of the warranty period, the owner shall assume the responsibility for the repair of the house and the cost of the repair. However, when there are safety problems in houses especially old houses, it is often difficult for developers and builders to find people who should take responsibility for this because of the long time. Therefore, compensation for housing maintenance is difficult to implement because of the problem with the capital, and many owners of older houses that are in urgent need of renovation do not have high economic income, so the cost of such renovation is still a burden to the owners.

## Conclusion

In this paper, we investigate residents’ cognition of housing security in Shanghai by an analysis of the attitude of housing users in the housing safety management process. The survey invited respondents to score a total of 10 building safety management questions on the questionnaire, where ranking rules were used to explore whether the safety awareness of old and new housing residents had significant agreements or disagreements and the potential factors influencing the safety consciousness were analyzed.

(1) The problem of housing security in Shanghai has become increasingly prominent; however the government management system is in its infancy, the policy norms are incomplete and the system is imperfect. Shanghai should learn from foreign advanced management experience and not only focus on the aftermath of the disaster but also improve the ability of predisaster early warning and prevention.

(2) Housing security awareness of Shanghai residents is at a medium level. The cognitive level of old housing residents’ housing security management consciousness is less than that of new housing residents. The three items of lowest scores are willingness to buy commercial housing insurance; willingness to use houses safely; and understanding of housing management law and policy. The current promotion of Shanghai housing security management policy is low, and cost is an influential factor in residents' safety consciousness. Therefore, Shanghai, which is in the process of building safety management, needs to intensify publicity to residents and expand multiple economic channels to solving safety problems.

(3) In this paper, three influence factors were obtained by principal factor analysis: asymmetric information, assessment of the safety status of the premises and differences in house users. Information asymmetry is the largest impact factor affecting Shanghai residents housing security consciousness, followed by the lack of information, the lack of housing security information, and the lack of housing policy management information. The lack of housing security to assess these factors limits the degree of residents' understanding of building management policy, such as repair and how to guarantee their legal rights. House safety evaluation factors indicate the safety problems that have occurred or will probably happen, which has some impact on the safety of the residents in use. The user status factor shows that residents' economic and cultural level has a certain impact on safety awareness. Therefore, it is necessary to strengthen the knowledge of residents. Every policy should comprehensively inform the residents to improve their own safety level.

Shanghai is the largest city in China, which is a developing country. This paper can provide a reference for other cities that need to carry out housing safety management. However, Shanghai has not suffered from obvious earthquakes and other geological disasters. Therefore, there is still the need to investigate those disasters to supplement the deficiencies that limit this study.

## Supporting information

S1 File Questionnaire survey in Chinese. (DOCX)

S2 File Questionnaire survey in English. (DOCX)
