# Supplementary material for: Evaluation of the factors influencing the housing safety awareness of residents in Shanghai
Source: PLoS One. 2020 Jan 24;15(1):e0227871. doi: 10.1371/journal.pone.0227871 (PMC6980500; doi:10.1371/journal.pone.0227871)
Supplement: S1 File — (DOCX) [file pone.0227871.s001.docx]

**上海市房屋管理政策市民接受度调查[复制]**

您好，此问卷主要针对上海市房屋管理的市民接受度进行调研，根据调研内容对政府房屋管理政策提出修改建议，希望答题者可以提供准确答案。本问卷所有问题不涉及个人隐私，并保证对问卷答案严格保密，不会给您带来困扰。本问卷将会占用您2-4分钟的时间，非常感谢您的参与！

1. 您的性别： [单选题] *

| ○男 | ○女 |  |  |  |  |  |  |
| --- | --- | --- | --- | --- | --- | --- | --- |

2. 您的年龄段： [单选题] *

| ○30以下 | ○30~45 | ○45~60 | ○60以上 |  |  |  |
| --- | --- | --- | --- | --- | --- | --- |

3. 您现所居住的房屋获得方式 [单选题] *

| ○拆迁分配 |
| --- |
| ○购买商品房 |
| ○购买单位公房 |
| ○单位分配 |
| ○租住国家或单位公房（宿舍等） |
| ○租住私房 |

4. 您房屋建成的时间 [单选题] *

| ○2010-2017年 |
| --- |
| ○2007-2010年 |
| ○1997-2006年 |
| ○1997年以前 |

5. 您是否考虑过采取拆墙等可能损害房屋安全的措施修改房屋格局 [单选题] *

| ○非常可能 | ○比较愿意 | ○会 | ○可能会 | ○非常不会 |
| --- | --- | --- | --- | --- |
|  | | | | |

6. 您在购买房屋或以后将要购买房屋时，是否考虑以下因素[矩阵量表题] *

|  | 完全不考虑 | 稍微考虑 | 考虑 | 比较重视 | 非常重视 |
| --- | --- | --- | --- | --- | --- |
| 房屋结构情况 | ○ | ○ | ○ | ○ | ○ |
| 房屋周边施工情况 | ○ | ○ | ○ | ○ | ○ |
| 房屋安全文书 | ○ | ○ | ○ | ○ | ○ |

7. 您是否能够接受政府等专业人员定期进入房屋内部进行安全检查？ [单选题] *

| ○非常不能接受 | ○比较愿意 | ○可以接受 | ○比较愿意接受 | ○非常愿意接受 |
| --- | --- | --- | --- | --- |

8. 您是否愿意自费对自己的住房进行定期检测 [单选题] *

| ○非常不能接受 | ○比较愿意 | ○可以接受 | ○比较愿意接受 | ○非常愿意接受 |
| --- | --- | --- | --- | --- |

9. 对房屋进行安全加固和修缮，您认为费用承担的组合方式应该是 [多选题] *

| □业主出钱 |
| --- |
| □政府补贴 |
| □购买商业保险 |
| □物业专项维修基金 |
| □其他 |

10. 如推出房屋安全商业保险，房屋出现非人为问题由保险公司进行理赔，您愿意购买的程度 [单选题] *

| ○完全不愿意购买 | ○考虑购买 | ○愿意 | ○比较愿意 | ○非常愿意购买 |
| --- | --- | --- | --- | --- |

11. 您对所在城市房屋管理的法律法规是否了解 [单选题] *

| ○不了解，并没有兴趣 |
| --- |
| ○不了解，但有兴趣 |
| ○了解一些，但没有兴趣 |
| ○了解一些，并有兴趣 |
| ○非常了解 |

12. 当您发现居住房屋发生安全问题时，会选择向以下哪些部门进行反映 [多选题] *

| □物业管理 | □居委会 |
| --- | --- |
| □房管局 | □城市规划管理局 |
| □城市管理行政执法局（城管） | □建设局（建委、建设办公室） |
| □其他 _________________ |  |

13. 如进行科普，您希望获取以下哪些信息[矩阵量表题] *

|  | 没兴趣 | 想要了解一些 | 想要了解 | 比较希望了解 | 急需了解 |
| --- | --- | --- | --- | --- | --- |
| 房屋问题如何判断及处理 | ○ | ○ | ○ | ○ | ○ |
| 房屋结构形式如承重墙柱等的布置 | ○ | ○ | ○ | ○ | ○ |
| 相关法律、法规等的宣讲 | ○ | ○ | ○ | ○ | ○ |
| 投诉、举报渠道 | ○ | ○ | ○ | ○ | ○ |

14. 如果通过房屋安全鉴定结果来限制房屋交易（如房屋安全度低将无法出售）您是否能够接受 [单选题] *

| ○能接受 |
| --- |
| ○不能 |

15. 您对于目前房屋安全管理还有哪些意见和建议（非必答题） [填空题]

_________________________________
