# Supplementary material for: Evaluation of the factors influencing the housing safety awareness of residents in Shanghai
Source: PLoS One. 2020 Jan 24;15(1):e0227871. doi: 10.1371/journal.pone.0227871 (PMC6980500; doi:10.1371/journal.pone.0227871)
Supplement: S2 File — (DOCX) [file pone.0227871.s002.docx]

**Citizen Acceptance Survey for Housing Management Policy in Shanghai**

This questionnaire is designed to collect data of citizen acceptance for housing management policy in Shanghai. Please provide your suggestion to the policy based on the survey and choose properly. All data collected will be confidential to prevent any personal privacy issues. You may need 2-4 minutes to finish the survey. Thanks for your cooperation!

1. Your Gender: (Single Choice) *

| ○Male | ○Female |  |  |  |  |  |  |
| --- | --- | --- | --- | --- | --- | --- | --- |

2. Your Age: (Single Choice) *

| ○Under 30 | ○30~45 | ○45~60 | ○Above 60 |  |  |  |
| --- | --- | --- | --- | --- | --- | --- |

3. What type is the house/apt you are living in now? (Single Choice) *

| ○Relocation Compensation |
| --- |
| ○Purchased as Commercial Housing |
| ○Purchased as Public Housing |
| ○Enterprise Distribution |
| ○Public Rental (such as dormitory) |
| ○Private Rental |

4. Your house/apt was built during (Single Choice) *

| ○2010-2017 |
| --- |
| ○2007-2010 |
| ○1997-2006 |
| ○Before 1997 |

5. Will you ever consider to change housing structure by measures such as demolishing the wall which could endanger the security of your house/apt? (Single Choice) *

| ○Much likely | ○Likely | ○Acceptable | ○Probably | ○Not likely |
| --- | --- | --- | --- | --- |
|  | | | | |

6. Will you ever take elements below into consideration when purchasing your house/apt?(Choose by Scale) *

|  | Not at all | Probably | Yes | It’s important | It’s very important |
| --- | --- | --- | --- | --- | --- |
| Housing Structure | ○ | ○ | ○ | ○ | ○ |
| Construction Situation near your House/Apt | ○ | ○ | ○ | ○ | ○ |
| Official document of your Housing Security | ○ | ○ | ○ | ○ | ○ |

7. Can you accept regular security examination by governmental agents in the house/apt?(Single Choice) *

| ○Impossible | ○Probably | ○Acceptable | ○受 Willing to | ○Very willing to |
| --- | --- | --- | --- | --- |

8. Are you willing to perform regular examination and appraising for your house/apt at your own expenses? (Single Choice) *

| ○Impossible | ○Probably | ○Acceptable | ○Willing to | ○Very Willing to |
| --- | --- | --- | --- | --- |

9. * In your opination, who shall pay for maintenance and consolidation projects for your house/apt? (Multiple Choice)

| □Owner of property |
| --- |
| □Government |
| □Commercial Insurance |
| □Real estate |
| □Others |

10. * If commercial insurance could settle a claim in case any non-artificial issues happen to your house/apt, are you willing to buy it? (Single Choice)

| ○Impossible | ○Probably | ○Acceptable | ○Willing to | ○Very willing to |
| --- | --- | --- | --- | --- |

11. Do you know about the housing policy in your city? (Single Choice) *

| ○No, I’m not interested. |
| --- |
| ○No, but I’m interested. |
| ○Yes, but I’m not interested. |
| ○Yes, and I’m interested. |
| ○I know it very well. |

12. What departments below are you going to reach when housing security issues happening? (Multiple Choice) *

| □Real estate Management | □Neighborhood Committee |
| --- | --- |
| □Real Estate Bureau | □Municipality on City Planning |
| □Urban Management | □Construction Bureau |
| □Others _________________ |  |

13. Which information do you want to know if it is available for you? (Choose by Scale) *

|  | Not interested | Probably | Interested | I would like to know | It’s necessary |
| --- | --- | --- | --- | --- | --- |
| How to check and deal with problmes in your house/apt | ○ | ○ | ○ | ○ | ○ |
| Housing Structure, such as deployment of bearing column. | ○ | ○ | ○ | ○ | ○ |
| Propaganda of Related Laws and Regulations | ○ | ○ | ○ | ○ | ○ |
| Approaches for Complaints and Reports | ○ | ○ | ○ | ○ | ○ |

14. Can you accept if housing market is restricted by result of security assessment? (For example, houses/apts with low security will be unable to be sold) (Single Choice)*

| ○Yes |
| --- |
| ○No |

15. Do you have any other suggestions or advices on housing security management? (You may skip this question)

_________________________________
